# Women’s Breastfeeding Support Experiences in Ireland: A Qualitative Framework Analysis

**DOI:** 10.1177/08903344251363597

**Published:** 2025-09-25

**Authors:** Niamh Mc Evoy, Elaine Lehane, Patricia Leahy-Warren, Rhona O’Connell, Liz Cogan, Michelle O’Driscoll, Helen Mulcahy

**Affiliations:** 1 The Health Service Executive, Dublin, Ireland; 2 Catherine McAuley School of Nursing and Midwifery, University College Cork, Cork, Ireland; 3 Pharmaceutical Care Research Group, School of Pharmacy, University College Cork, Cork, Ireland

**Keywords:** breastfeeding, breastfeeding support, diary entries, healthcare workers, maternal support, mothers’ experiences, qualitative designs

## Abstract

**Background:**

Breastfeeding rates in Ireland are among the lowest in the world. Social support networks influence initiation and duration.

**Research Aim:**

To describe pregnant and postpartum women’s encounters and experiences with health care professionals, family, or friends around breastfeeding in Ireland.

**Method:**

Using a qualitative framework design, data were collected as part of a wider study. Online diary entries were collected between October 2021 and May 2022. Participants reflected on their breastfeeding encounters, focusing on timing, context, and feelings evoked about breastfeeding support. These a priori categories were the starting point for analysis.

**Results:**

Participants (*N* = 27) produced a total of 91 diary entries entered while participants were from 13 weeks pregnant to 30 weeks postpartum. Most interactions recorded were with family members, friends, and lactation support providers. Three themes were identified: (1) Family Circle of Influence, (2) It Takes a Village, and (3) Maternity Service Providers. “Family Circle of Influence” included reflections on the influence of those closest to participants on their breastfeeding journey. Partners and female relatives were generally supportive and provided emotional support, despite having concerns about the decision to breastfeed. “It Takes a Village” covered troubleshooting feeding issues with friends and support groups. Experiences within this wider community group were influential, positively and negatively. “Maternity Service Providers” captured information sources, skills provision, and conflicting advice, which sometimes undermined maternal confidence.

**Conclusion:**

Participants’ breastfeeding journeys included encounters with family, social networks, and wider health services. Cumulative diary entries provided reflections on the emotional impact of supportive or undermining interactions on breastfeeding decisions.

## Background

Breastfeeding is widely acknowledged as the best method of nutrition for optimal development and growth of infants (Purdy et al., 2017; World Health Organization [WHO], 2021). When healthcare personnel (HCPs) provide high-quality support for breastfeeding with appropriate information and positive attitudes, enhanced exclusivity and longer duration of breastfeeding can occur (Gerhardsson et al., 2023), as well as improved maternal mental health outcomes (Yuen et al., 2022). Breastfeeding support can be provided throughout pregnancy and postpartum at the individual or group level.

The breastfeeding initiation rate (i.e., the proportion of infants who were ever breastfed) in Ireland was 63% in 2022 (Health Service Executive [HSE], 2023a). This is relatively low compared to 83% of newborns ever breastfed in the United States (U.S. Centers for Disease Control and Prevention [U.S. CDC], 2022), and 78.8% in high-income countries overall (United Nations Children’s Fund [UNICEF], 2018). The WHO recommends exclusive breastfeeding for the first 6 months of life and has set a global target for 50% of infants worldwide to be exclusively breastfed at 6 months by 2025. In Ireland, among infants assessed during the Public Health Nurse’s first home visit (within 72 hours of discharge from a maternity unit), 62% had ever been breastfed in 2022 (HSE, 2023b). When examining any breastfeeding at 3 months of age, the proportion increased from 35% in 2015 to 42.3% in 2019, indicating a positive trend (HSE, 2021). However, exclusive breastfeeding to 6 months remains very low, as less than 6% of babies who commenced breastfeeding were exclusively breastfed up to 6 months of age (HSE, 2021). This figure reflects a cohort of infants followed from birth to 6 months, not a cross-sectional snapshot. The World Breastfeeding Trends initiative WBTi Ireland (2023) report acknowledged that improvements in breastfeeding rates over the past decade are linked to strengthened policies and governance structures supporting breastfeeding. However, challenges with sustaining breastfeeding—particularly exclusive breastfeeding—persist in Ireland, and are also seen globally. Addressing these challenges requires broad societal efforts to improve both breastfeeding initiation and continuation rates at the population level (Pérez-Escamilla et al., 2023)

At the national level, Healthy Ireland (2019) is a government-led initiative that has clear goals and an Implementation plan with linked actions (HSE, 2023b). One of the actions is for a named lead health program to increase infant feeding supports and increase infant feeding awareness across health, education, and local authority sectors. Exclusivity and longevity of breastfeeding are improved when support is accessible via trained peer supporters or healthcare professionals. For instance, in Ireland, ongoing visits are scheduled or planned so that women can predict when support will be available. The goal is that the support provided is individualized to the needs of the population it serves (McFadden et al., 2017). Partnerships between public health sectors and businesses are encouraged by the HSE (2023a) to promote health and well-being, such as a more breastfeeding-friendly environment in local communities. These initiatives do not have a financial incentive but are considered to benefit public health. However, a lack of breastfeeding experience and population support in community settings in Ireland is an ongoing concern and slows the achievement of the Healthy Ireland vision (Leahy-Warren et al., 2017; Philip et al., 2023; WBTi Ireland, 2023).

**Key Messages**

- Researching women’s perinatal journey across hospital and community helps us understand perceptions of the influence of social and healthcare encounters on support for breastfeeding.
- Participant diaries provided insight into how interactions with family, friends, healthcare providers, and the wider community affected individual decision-making, and the initiation and continuation of breastfeeding.
- Some maternal participants in our study actively sought support from healthcare providers; however, there were also reports of receiving unsolicited advice or experiencing negative interactions in healthcare settings, leaving participants feeling uncertain about breastfeeding.

Breastfeeding is influenced by multiple cultural, psychological, and physiological factors (Lyons et al., 2021). Mothers can seek breastfeeding support from various sources and close networks, including family, friends and partners, healthcare providers, lactation-specific healthcare providers, and other community members. Family, friends, and partners play the most influential role in assisting mothers to accomplish their breastfeeding goals (Laugen et al., 2016). This paper is part of a larger study entitled Practice Enhancement for Exclusive Breastfeeding (PEEB) across a woman’s perinatal journey, including hospital, general practitioner (GP) settings, and primary health care locations (staffed by public health sector professionals; Mulcahy et al., 2025). In Ireland, there is little research from the perspective of mothers regarding their perceptions of the quality of support they receive and how these supports operate over time (Hannon et al., 2022). The current study aims to explore women’s pregnancy and postpartum experiences in Ireland during their perinatal breastfeeding encounters with family, friends, partners, healthcare professionals, and other community members, using guided diary entries.

## Methods

### Design

This prospective, observational analysis, which is part of the Practice Enhancement for Exclusive Breastfeeding (PEEB) study (Mulcahy et al., 2025), involved asking maternal participants to create online diary entries describing their feelings about interactions that influenced or impacted their breastfeeding journey. In this study, diaries were used to collect data that could provide insight into participants’ experiences over time. Diary entries allowed the researchers to examine participants’ experiences in their natural, spontaneous context, rather than retrospectively, reducing the risk of recall bias by minimizing the time between their experience and their account of it (Bolger et al., 2003). These insights may not have been possible to uncover through interviews, a common method for data collection, which can be influenced by social desirability and only captures a single moment in time (Hinsliff-Smith and Spencer, 2016). Ethical approval was obtained from the university social research ethics committee (ECM 4 [ee] 10/8/2021 and ECM 3 [c] 19/10/2021).

#### Setting and relevant context

The study occurred in Ireland, a high-income country with a well-educated population and free maternity care in the public health system. Midwives and public health nurses are the primary providers of hospital and community breastfeeding support. Midwives in the hospital also provide individual and group breastfeeding educational sessions and facilitate individual consultations and peer support groups. On hospital discharge following a 2 to 3 day stay, community peer support groups are facilitated by Public Health Nurses and International Board-Certified Lactation Consultants (IBCLC). IBCLCs are employed by the public health services to provide lactation support but, at the time of this study, their availability to provide breastfeeding support was limited due to unfilled staff positions. There were also staff shortages in other public health positions, which likely limited the capacity of healthcare professionals generally to provide breastfeeding-related support to women (Griffin, 2022). Outside of the public health system, women can also access volunteer peer lactation services through La Leche League of Ireland, among other support groups, as well as informal online or tele-lactation peer support resources, and private lactation consulting services.

#### Sample

We used convenience sampling to recruit eligible participants at one hospital site in the south of Ireland. At the antenatal booking visit at 12 weeks of gestation, participants were screened for eligibility criteria. The only inclusion criterion was their intent to breastfeed. Prospective participants were contacted by the booking midwife (clinical staff) for participation in the full study. Those who could not read or write in English were ineligible to participate. Compensation was not provided.

A total of 86 women consented to participate in the diary entry part of the main study, and 27 participants completed at least one diary entry. The reasons why 59 selected people chose not to respond are unknown. From reviewing relevant literature (Parkinson et al., 2016; Schol et al., 2024), a sample size of 20 to 40 is typical for similar studies, but additional efforts are required to avoid loss of participants over time. While the overall depth of data is an important consideration, engagement ultimately depends on participant willingness, in addition to researcher encouragement.

#### Data collection

Data were collected from October 2021 to May 2022. Participants who indicated their interest in the study provided their email address and were sent an introductory email by research staff with information and instructions about how to submit the online diary entries. Participants who returned the signed consent form by mail (*N* = 86) were sent details about how to post diary entries via a reusable link (electronic) hosted on Google Forms. Data could be submitted any time during pregnancy and up to 3 months postpartum.

Participants were asked to record any interactions that influenced their breastfeeding journey: who they encountered, what was said, and what their feelings about any interaction were, such as reassurance, empowerment, uncertainty, confusion, anger, or any other experience they wished to record. They could indicate whether the interactions were very positive, positive, neutral, negative, or very negative. However, these were only suggested instructions, and participants were free to record text in whichever way they wished. The context of the interaction was not requested. Participants created diary entries wherever and in whatever context they chose, using a private pseudonym so that multiple submissions could be linked from the same participants on the online diary while protecting their identity. Participants completing diary entries were not asked for their age, marital status, or level of education. At 36 weeks of pregnancy 93.1% (*n* = 54) of the full study sample still intended to breastfeed. Whereas at six 6 postpartum (T3), 57.1% (16 of 28 who responded) were exclusively breastfeeding, and 32.1% (*n* = 9 of 28) reported to be partially breastfeeding their baby. At 3 months postpartum, 44.6% (*n* = 29 of 65) were exclusively breastfeeding, 24.6% (*n* = 16 of 65) reported to be partially breastfeeding, and 30.8% (*n* = 20 of 65) were formula feeding

The expected date of delivery was collected at the commencement of the diary entry to facilitate reminder emails, which were sent at 32 weeks' gestation and 2 weeks postpartum. Reminder posters were also placed in the maternity waiting areas and on the information screen at the hospital entrance. At the end of the study, a final email was sent to all participants, thanking them for their engagement and informing them about when the diary link would close. All data were anonymized and saved in one folder as a password-protected file, which was securely stored. Access to the collected data was limited to researchers involved in its analysis.

#### Data analysis

Diary entries were analyzed using Qualitative Framework Analysis (Ritchie & Spencer, 2002; Snowden, 2015). Framework Analysis was chosen because it is well-suited for large qualitative data sets (Baldwin, 2021; Goldsmith, 2021) and allowed for examination of data as it changed with participant entries. Additionally, it ensured a robust audit trail throughout the stages of analysis. Analysis followed the stages of Framework Analysis: (1) data familiarization; (2) identifying a thematic framework; (3) indexing all study data against the framework; (4) charting to summarize the indexed data; and (5) mapping and interpretation (Goldsmith, 2021).

The first coder read diary entries multiple times and considered their context and timing (Stage 1). Next, the framework was identified through a combination of a priori categories and themes identified during data familiarization. Entries were visualized for each individual participant and across participants for recurring words and phrases, cognizant of the a priori categories, and then data-driven themes were identified (Stage 2). The a priori categories were the timing, who the encounter was with, feelings evoked, and any other experiences. Diary entry data were assigned category labels using Microsoft Excel (Stage 3). Despite initial suggestions about gradients of negativity and positivity, all entries were free text. Thus, when the data were being analyzed, a positive, negative, or neutral color coding was applied to each entry, aligned with the tone of the interaction. The color coding process allowed for clarity as participants’ views remained connected to their accounts within the themed matrix and thus individual viewpoints were not lost (Collaço et al., 2021). The developing framework was reviewed and discussed by three researchers (NMcE, RO’C, and HM; Stage 4), and a further matrix captured the recurring themes. Overarching labels were applied by consensus to the abstracted themes (Stage 5).

To enhance rigor, three researchers independently reviewed each stage of data analysis, and any conflicts were resolved by discussion and consensus. The primary researcher had no prior relationship with participants. In this study, the novice researcher (a public health nurse undertaking a master's program) worked under two supervisors (with PhD qualifications). Both supervisors independently reviewed the analysis conducted by the primary researcher. Analysis was conducted using Microsoft Excel (Stages 1–4) and Microsoft Word (Stage 5) and SPSS (Version 27).

## Results

### Participant Characteristics

Participants in this study were recruited from within the larger study, and no demographic data on age, marital status, education, or other demographic data were collected. Participants (*N* = 27) made a total of 91 diary entries, from 13 weeks antenatal through 30 weeks postpartum. The mean number of entries per participant was three (range 1–24), and the median was one entry per participant. Some participants provided antenatal entries only (*n* = 16, 60%). Only nine participants (33%) provided postpartum data, with just two (7%) contributing data in both the antenatal and postpartum periods. The participant who inputted the most entries across both periods reported breastfeeding up to 7 months postpartum. The weeks in which the most responses were recorded during pregnancy were 33 weeks (*n* = 14, 15%) and 34 weeks (*n* =10, 11%), which could be attributed to the reminder sent at 32 weeks of gestation.

Participants reported diverse exchanges with an array of people in a variety of locations. Most of the interactions were with family members and friends, followed by health care professionals and lactation support providers (Table 1).

**Table 1.** Participant Interaction Involving Breastfeeding With Different People Noted in Diary Entries (*N* = 27 Participants, *N* = 91 Diary Entries).

Persons Mentioned in the Diary Entry With Whom the Participant Had Interactions	Number of Mentions Within Diary Entries (%)	Number of Participants (%) Who Made the Mentions
Family Member	21 (23)	10 (37)
Partner	5 (5.5)	5 (1.5)
Friend	19 (21)	10 (37)
General Practitioner	2 (2.1)	2 (7)
Nurse	2 (2.1)	1 (4)
GP Practice Nurse	3 (3.2)	1 (4)
Public Health Nurse	3 (3.2)	3 (11)
Midwife	11 (12)	8 (30)
IBCLC	10 (11)	7 (26)
Breastfeeding Advisor/Community Groups	3 (3.2)	2 (7)
Other (shopkeeper, member of public, yoga instructor)	11 (12)	6 (22)
Other Healthcare Center staff member (porter, cleaner, receptionist, catering staff etc.)	1 (1)	1 (4)
	91 (100%)	*N* = 27 (100%)

*Note*. GP = general practitioner; IBCLC = International Board Certified Lactation Consultant

**Figure 1.**
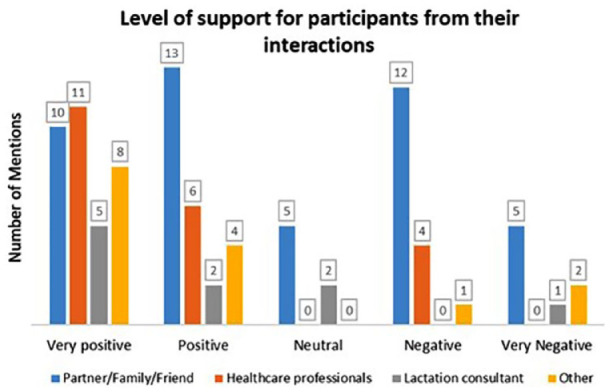
Categorization of Interactions.

Interactions with family members and friends were more frequently reported than those with lactation support providers or community groups. Table 1 describes the number of interactions and with whom those interactions occurred. Face-to-face interactions were most common (*n* = 73, 80% entries from 24 [89%] participants), followed by online (*n* = 9, 11% entries from five [19%] participants), and phone (*n* = 8, 9% entries from six [22%] participants), and one other participant recorded one reflective entry unrelated to an interaction. The comments were categorized according to how participants felt about interactions and their impact (Figure 1).

There was wide variation in how participants reported the impact of interactions. They mainly reported positive interactions with health care workers, family, and friends, but there were also negative comments. Most negative comments came from casual encounters, including people the participants met in shops or at events.

Further analysis identified three themes, which were: (1) Family Circle of Influence; (2) It Takes a Village; and (3) Maternity Service Providers.

#### Theme 1: Family Circle of Influence

The focus of this theme was participants’ interactions with family members, and it was considered important in terms of how influential these interactions were on their breastfeeding journeys. They emphasized the support of those closest to them and were influenced either positively or negatively by these interactions. No conversations with male family members besides partners were recorded in the diary entries.

##### Subtheme 1: Interactions with partners

Partners being supportive of a plan to breastfeed was an important factor in the participants’ decisions to breastfeed. Conversations with partners taking place at home were documented in 17 of the 91 (19%) diary entries. Partners were generally seen as supportive at the outset of the participant’s decision to breastfeed. However, the participant reported that their partners also communicated their own fears and anxieties during the pregnancy about breastfeeding, particularly in relation to the functioning of the family unit and the impact on the participant’s mental health. Julie, at 33 weeks pregnant, described a conversation with her partner after a breastfeeding class: “He is very supportive of my decision to breastfeed. He is worried, though, that it will have a negative impact on my mental health due to previous postpartum anxiety and depression.” Julie elaborated that this left her feeling apprehensive about her breastfeeding future. She commented that she had no family members with breastfeeding experience and considered that she might not get much help because of this, during her postpartum period. Thus, she did not feel the need for family interactions about breastfeeding during the postpartum period.

##### Subtheme 2: Interactions with female relatives

Interactions with female relatives happened antenatally and postpartum and were mostly spontaneous conversations with sisters, mothers, and mothers-in-law. Participants did not receive support from all their relatives but stated that there were some available to “lean on” if support was lacking from others. Interactions reported were both positive (*n* = 6) and negative (*n* = 7) in nature.

Positive interactions left participants feeling empowered, reassured, and more confident in their plans to breastfeed, as they received both practical and emotional support from some female relatives. A participant who breastfed for 8 months, Lara, at 32 weeks pregnant, wrote that she had support from her own mother and other mothers. Wilma described a positive interaction with her mother-in-law at 33 weeks pregnant: “[She] inquired if I intended to breastfeed this baby. I said yes, and she told me about the four experiences she had with her children, and if there was anything she could do to help, just ask.”

Conversely, some participants reported that their choice to breastfeed conflicted with the views of maternal figures in their lives. Negative comments eroded confidence in their breastfeeding decision, leaving them feeling isolated and judged. Jennifer, at 32 weeks pregnant, described an incident that happened in her home with her mother-in-law. “When I talk about breastfeeding and my plan for it, she [mother-in-law] rolls her eyes at me and says I’m full of notions”. Jennifer wrote that this interaction left her feeling annoyed but more determined. Christine recorded her own mother’s discouraging comment to her at 7 weeks postpartum: “[My mother] said to me that she doesn’t know anything about [breastfeeding] as they didn’t do it in her time.” Christine did not provide any reflective comment about this.

#### Theme 2: It Takes a Village

The support or discouragement that participants experienced in relation to their breastfeeding journeys extended beyond their family to a wider network of individuals outside the healthcare delivery system. This scaffolding of friends, breastfeeding support groups, and work colleagues was a community of connected people in proximity and potentially available to talk with the participant.

##### Subtheme 1: Friends

In total, 19 out of 91 (21%) interactions were with friends, mostly spontaneous in nature, which provided tangible support for them to breastfeed. Participants generally reported that their friends were proactive and forward-thinking in problem-solving and troubleshooting around breastfeeding issues, supporting them in a way that their own partners could not necessarily do. This practical help and direct involvement served to enhance the emotional support that the participants experienced. At 17 weeks pregnant, Mary described an interaction with a friend, who promised to help find answers to her breastfeeding questions, which left her feeling more confident. Grainne reported conversations she had with a friend while walking in a park with her infant (12 weeks postpartum) in which they discussed how to solve some of the ongoing feeding issues. For example, “We both saw the baby was teething and she was in a lot of pain. I gave her paracetamol, and the baby breastfed with no problem.” This was a helpful and practical suggestion in which they were both able to discuss the teething problem and come to a decision about what medication to use. This interaction enhanced Grainne’s confidence that she was able to respond to her baby’s cues.

Some friends negatively influenced the participants’ views of breastfeeding. Participants were made aware of the struggles their friends had when breastfeeding. Nadine, at 36 weeks pregnant, reported an interaction with a friend who was breastfeeding her 4-week-old baby and experiencing difficulties. For Nadine, breastfeeding seemed to be “a lot harder mentally” than she had anticipated. Other participants recounted similar negative breastfeeding stories from friends and acquaintances. For some, this was disheartening, but for others, it made them more determined to succeed.

##### Subtheme 2: Support groups

Participants sought and received support both in-person and online from support groups. These were mostly postpartum support groups. They reflected on the community breastfeeding support (e.g., community group, PHN, and practice nurse) in a positive light (*n* = 6, participants; nine interactions), and these interactions were perceived to increase confidence in both breastfeeding skill and the decision to breastfeed. Grainne received reassurance and advice from attending a PHN facilitated support group, where she felt “understood.” Mary revealed that as she did not have a local support group, she sought out support from online groups. Most participants spoke about obtaining practical knowledge, techniques, and advice from attending these groups. Grainne reported that hearing other support group attendees’ stories prepared her to be assertive in her breastfeeding decision.

##### Subtheme 3: Work colleagues

Returning to work while breastfeeding featured in the diary entries. Colleagues attempted to provide support, but their prior experiences tended to center on their own negative anecdotes and fears. Grainne reported hearing from a work colleague about how she had to “pump next to smelly shoes” and, to her, this implied that breastfeeding was not valued in her own work environment. Wilma reported a colleague discussing their fear of breastfeeding in front of others, which echoed an existing fear of her own: “When I said breastfeeding, she said she would have loved to but was too nervous to do it in front of anybody else. This is a fear I have myself.” One participant disclosed in her diary entry that she breastfed her previous baby for just 3 weeks. She commented that this was influenced by “lots of negative discussion from work colleagues related to breastfeeding.” However, she reported she was more positive about breastfeeding during her index pregnancy.

#### Theme 3: Maternity service providers

While Themes 1 and 2 focus on the individual’s own resources of family, friends, and wider networks, Theme 3 highlights the significant role of the maternity service providers in supporting women in their breastfeeding journey, specifically in relation to the provision of information, skills, and encouragement. The majority of interactions reported were with HCPs, and it was evident from the narrative that the participants made an appointment to meet the HCP rather than waiting for an interaction to happen by chance.

##### Subtheme 1: Information and skills

Participants sought information and help with practical skills from midwives and PHNs whom they encountered during pregnancy and postpartum. Breastfeeding classes were either provided by the maternity services or sourced separately, and were mostly delivered by IBCLCs. These services were deemed useful by participants. However, some felt that too much information overwhelmed them, and a balance needed to be struck. PHNs were reported to be forthcoming with useful advice on practical issues such as managing nipple pain or travel advice.

Some participants reported receiving conflicting information from HCPs, which appeared to impact them negatively. This served to confuse and worry. As Christine said, “It just made me question why, even in the medical profession, breastfeeding information is ambiguous and people tell you a different thing,” and thus further information needed to be sought. Christine shared these thoughts with a friend in the postpartum period, who told her: “Staff in hospital giving conflicting evidence about breastfeeding after labour are no help and [my friend] needed to pay privately to get [a lactation support provider] to call to the house.”

Diary entries reported the important role that HCPs play in the development of breastfeeding skills. Bethany reflected on a positive experience: “The kind midwife showed me how to breastfeed lying down at nighttime. . . . I appreciated this support from the midwife.” This interaction left Bethany feeling supported; she had considered stopping breastfeeding, but then managed to continue for 2 months postpartum.

Colostrum harvesting was an example of a specific skill that caused anxiety and nervousness amongst participants and featured in nine of the 91 diary entries. Participants reported feeling overwhelmed, uncertain, and confused regarding the process from early pregnancy. At an antenatal class, Christine’s midwife advised the “opposite of what the IBCLC recommended” about the importance of colostrum harvesting. At 39 weeks of pregnancy Christine reported, “I started collecting colostrum from Week 36 on. I found the process very stressful. . . . I decided to take a break from collecting for a week. It was making me nervous about breastfeeding.” Participants described helpful interactions with midwives when mentioning colostrum harvesting, but they also discussed barriers in finding information and using YouTube to figure it out. Specific skills training would have been welcomed in this instance.

##### Subtheme 2: Encouragement and reassurance

Lack of encouragement, or active discouragement, could be detrimental to a participant's breastfeeding experience. Christine described an interaction with a health care assistant in the hospital postnatal ward:

[The health care assistant] spent the day telling me the baby was starving and to give him a bottle of formula. I eventually gave in. . . . My partner told me later that she gave everyone in the ward a bottle that day, and all of us were breastfeeding.

Christine felt angry and confused, and with her confidence undermined, she became unsure if her baby was hungry or not. The uncertainty that participants experienced during their breastfeeding journey was often paralyzing, and they relied heavily on HCPs to reassure and encourage them and to provide that emotional support. At 33 weeks, Julie discussed her experience of attending an online breastfeeding course and finding it to be a “very reassuring interaction,” illustrating that her needs were met from outside the care of maternity providers.

## Discussion

The current study examined 91 diary entries among 27 participants between 13 weeks of gestation through 30 weeks postpartum and analyzed experiences of interactions with personal and professional connections whilst on the perinatal breastfeeding journey. Participants’ entries represented a compilation of unfiltered data on both the protective and impeding factors encountered. The findings showed that participants engaged with family, friends, and HCPs in a manner that impacted their decisions to commence, continue, and persevere with breastfeeding in the face of challenges. Despite prompting, not all participants provided entries during the antenatal or postpartum period, and, therefore, these participants’ experiences remain unknown. From the three themes identified in the study, family interactions primarily occurred with partners and female relatives. Participants in this study reported that partners were supportive of their breastfeeding decision. This finding was corroborated in a study by Budiati et al. (2022) of husbands (*n* = 12) of women who delivered by Caesarean section living in West Java. These husbands were knowledgeable about the benefits of breastfeeding, and all committed to supporting their wives to breastfeed. Using a small sample of Chinese mothers (*n* = 14) living in Ireland, Zhou et al. (2020) reported that supportive partners or family were conducive to successful and longer duration of breastfeeding. Participants considered that the absence of this assistance reduced the duration of breastfeeding, as without these family members and partners, the participants lacked sufficient practical support and time for recovery and lactation to be well established. The findings in the current study demonstrated that partners were open to breastfeeding conversations, but their overriding concern was for participants’ mental health and well-being. This is a similar finding to a qualitative systematic review (Earle & Hadley, 2018) comprising 20 studies across different countries (e.g., the United Kingdom and the United States), which found that men tended to leave breastfeeding decision-making to women and viewed their role as mainly supportive.

Participants in this study reported experiencing varying levels of support from female relatives, which contributed to feelings of either empowerment or isolation. A qualitative study based on 30 mothers in New Zealand (Alianmoghaddam et al., 2018) assessed the influence of being able to connect with family using social media on exclusive breastfeeding practice. Participants accessed breastfeeding support through the internet from family members who were far away. They accessed practical and emotional breastfeeding support from them via Skype. From the research findings, when practical and emotional family breastfeeding support is part of the culture within a family, it boosts maternal empowerment.

In our study, it was observed that female figures (including mothers, mothers-in-law, sisters, and aunts) were reported as influential to varying degrees. When participants were encouraged by their friends to troubleshoot breastfeeding problems or to discuss the challenges they were experiencing, they felt supported and understood. There were also other participants in the study who reported negative interactions with their friends, but in the absence of further entries, the impact is unknown. Nevertheless, there is strong evidence that breastfeeding rates and duration in the United States correlated with information from family/friends and breastfeeding support groups (Quintero et al., 2023).

Group support for participants was positively reported in this study, indicating that participants who attend such groups found support and social connection amongst their peers, corroborating findings from previous research (Quinn et al., 2019). In addition, attendance at breastfeeding support groups can facilitate participants to feel less marginalized in their decision to breastfeed (Jackson & Hallam, 2021; Leahy-Warren et al., 2017). The latter study identified the value of feeling supported in a like-minded group of breastfeeding individuals, in what was otherwise described as a primary bottle-feeding culture.

Participants’ encounters with their colleagues generally did not leave a positive impression. This was primarily due to the reported negative breastfeeding experiences of their colleagues. Although women in Ireland have been entitled to breastfeed or express breastmilk in the workplace since 2004 (HSE, 2023b), the reality is that this policy is poorly implemented (Desmond & Meaney, 2016). International evidence supports the need for adequate environmental supports, such as adequate lactation rooms, to motivate continuation of breastfeeding upon returning to the workplace (WBTi-Ireland, 2023; Yu et al., 2023). The cost implications for businesses cannot be underestimated, and incentivization to create or build appropriate spaces, as well as offering breastfeeding resources or better remote working options, should be considered.

While some participants did actively seek breastfeeding support from HCPs or ask breastfeeding-related questions, there were concerning instances described of unsolicited advice or negative interactions. On occasions when negative interactions with HCPs were reported, the situation was salvaged by another HCP. Provision of infant formula to solve a breastfeeding problem undermines breastfeeding efforts and can be discouraging to those who opt to breastfeed (O’Sullivan et al., 2021). HCP breastfeeding support is fundamental to aligning expectations with reality, reassurance, and assisting mothers to learn new skills (Edwards et al., 2017; Fraser et al., 2020). Guidance and technical skills from HCPs are highly appreciated by breastfeeding mothers (Cook et al., 2021). Thus, it is important that breastfeeding education for healthcare providers requires training in practical breastfeeding skills in addition to theoretical training (Mulcahy et al., 2022). Conflicting advice in relation to breastfeeding is pervasive (Leahy-Warren et al., 2017), and the current findings highlight the need to focus on evidence-based interpersonal and solution-focused interactions (Gavine et al., 2022). One Irish study reported that consultation with a lactation support provider during postnatal hospitalization, in addition to the support of a partner and general postnatal support were the three most influential factors in women’s decision to initiate and/or continue breastfeeding (Alberdi et al., 2018). The lack of breastfeeding support from the healthcare system during the postpartum period is well recognized in Ireland (Lawlor et al., 2023).

## Limitations

Several limitations should be noted when considering our findings. The age, gender, and level of education are not known for participants providing diary entries, and the non-probability sample was confined to one geographical area of Ireland. The sample may not have included participants from non-traditional and/or LGBTQ families. The study did not follow up with those who stopped diary entries, and it was not established if these participants were successful with their breastfeeding goals. Reminders were sent to maximize participation, but it must be acknowledged that depth of data was not achieved.

## Conclusion

Participant diaries are a novel data collection approach in breastfeeding. The results provide greater insight into the impacts that interactions with family, friends, and the wider community have on decision-making, initiating, and continuing breastfeeding over time. Further research to collect more detailed and reflective diary entries may have the potential to capture greater cultural nuances that could enhance targeted interventions at both the individual and community level.
